# News and Notes

**Published:** 1915-06

**Authors:** 


### NEWS AND NOTES

### PERSONAL.

Dr. E. P. Merritt, who has been associated for several years with Dr. W. L. Champion, has opened offices of his own at 924-5-6 Candler Building, Atlanta. His work will be limited to the disease and Surgery of the Genito-Urinary System.

### FIFTEENTH ANNUAL SESSION OF THE CHATTAHOOCHEE VALLEY MEDICAL AND SURGICAL ASSOCIATION.

To be Held in Opelika, Alabama, July 13th and 14th, 1915. Place of Meeting: Elks' Home.

PROGRAM:

Call to order 10 a. m.

Invocation—Rev. W. P. Hines, Opelika, Ala.

Address of Welcome in behalf of the City of Opelika—By M. M. McCall, President Chamber of Commerce.

Address of Welcome in behalf of Lee County Medical Society—By Dr. G. H. Moore, Opelika, Ala.

Response in behalf of the C. V. M. & S. A.—By Dr. J. H. McDuffie, Columbus, Ga.

1. Arterio Sclerosis—By J. M. Poer, M. D., West Point, Ga. Leaders in discussion, S. R. Roberts, J. M. Welch, B. L. Wyman.

2. Vital and Mortuary Statistics—By G. H. Perry, M. D., Montgomery, Ala. Leaders in discussion, H. W. Terrell, W. W. Stevenson, J. N. Baker.

Afternoon Session, 2 p. m.

3. The Blood—By C. W. Gould, M. D., Atlanta, Ga. Leaders in discussion, James S. McLester, E. C. Thrash.

4. Gastro-Intestinal Toxemia—By C. C. Farguson, M. D., Camp Hill, Ala. Leaders in discussion, Geo. M. Niles, G. P. Huguley, Willis Jones.

5. The Fallacy of Diagnosing Ureteral Calculi, with report of cases to illustrate—By Willis Jones, M. D., Atlanta, Ga. Leaders in discussion, M. L. Boyd, Leon Watkins.

6. Infectious Diarrhoea of Infants—By W. W. Harper, M. D., Selma, Ala. Leaders in discussion, Geo. M. Niles, Gaston Griel.

7. Major Operations Under Local Anaesthesia—By W. A. Selman, M. D., Atlanta, Ga. Leaders in discussion, H. P. Cole, W. P. Nicolson, E. G. Jones.

8. Some Latent Manifestations of Malaria—By H. W. Terrell, M. D., LaGrange, Ga. Leaders in discussion, Stewart R. Roberts, Thos. F. Taylor, E. C. Thrash.

8. Esophagitis—By Stewart R. Roberts, M. D., Atlanta, Ga. Leaders in discussion, W. W. Harper, J. C. Johnson.

9. Intestinal Obstruction—By W. C. Gewin, M. D., Birmingham, Ala. Leaders in discussion, L. C. Fischer, W. L. Cooke, Willis Jones.

10. Intussusception, Report of a Case—By A. L. Harlan, M. D., Alexander City, Ala. Leaders in discussion, R. R. Kime, Jas. L. Campbell.

11. Pellagra—By Geo. C. Mizell, M. D., Atlanta, Ga. Leaders in discussion, Geo. M. Niles, J. H. McDuffie, R. T. Dorsey.

12. The Treatment of the Psycho Neuroses Following Artificial Menopause—By B. L. Wyman, M. D., Birmingham, Ala. Leaders in discussion, Hansell Crenshaw, W. F. Shallenberger, E. G. Jones.

13. Surgery in the Home—By J. W. Alsobrooks, M. D., Plant City, Fla. Leaders in discussion, J. A. Goggans, N. A. Wheeler.

14. The Therapeutic Value of the Neisser, Antitoxin—By J. W. Clark, M. D., Columbus, Ga. Leaders in discussion, W. L. Champion, M. L. Boyd.

SECOND DAY—MORNING SESSION, 8:30 A. M.

15. Report of a Case of Double Uterus—By W. T. Langley, M. D., Camp Hill, Ala. Leaders in discussion, T. Brannon Hubbard, H. S. Munroe.

16. Uterine Curettage, Its Uses and Dangers—By W. F. Shallenberger, M. D., Atlanta, Ga. Leaders in discussion, J. C. Wooldridge, F. K. Boland.

17. Intestinal Parasites in Children—By Gaston Griel, M. D., Montgomery, Ala. Leaders in discussion, W. W. Harper, A. P. Griffith, R. T. Dorsey.

18. Treatment of Soiled Wounds by Organization of Blood Clots Under Wet Antiseptic Dressing—By W. P. Nicolson, M. D., Atlanta, Ga. Leaders in discussion, W. F. Westmoreland, W. C. Gewin.

19. When is Malaria Cured?—By R. J. O'Neal, M. D., West Point, Ga. Leaders in discussion, H. T. Hamner, H. W. Terrell.

20. Cirrhosis of the Liver—By C. E. Williams, M. D., Notasulga, Ala. Leaders in discussion, R. R. Kime, C. C. Farguson, J. M. Poer.

21. The Use of Morphine and Scopalamine in Labor—A Clinical Study—By J L. Campbell, M. D., Atlanta, Ga. Leaders in discussion, W. F. Shallenberger, T. Brannon Hubbard, B. L. Wyman.

22. The Use of the Percy Cautery in the Treatment of Inoperable Carcinoma of the Uterus—By J. D. S. Davis, M. D., Birmingham, Ala. Leaders in discussion, L. W. Morris, E. G. Jones.

23. Some Indications for Ceasarian Section, with Report of Cases—By Isham Kimbell, M. D., Auburn, Ala. Leaders in discussion, Hugh McCulloh, F. K. Boland.

24. Wassermann Values—By E. C. Thrash, M. D., Atlanta, Ga. Leaders in discsusion, Jas. McLester, C. W. Gould, J. W. Clark.

25. Tuberculosis in Country Practice—By J. O. Griffin, M. D., Alexander City, Ala. Leaders in discussion, J. M. Welch, W. W. Stevenson, H. T. Hamner.

26. The Value of Emetine in the Treatment of Pyorrhea Alveolaris—By Wm. Crenshaw, D. D. S., Atlanta, Ga. Leaders in discussion, E. C. Thrash, V. W. Heard, H. B. Peacock.

Afternoon Session—Second Day, 2 p. m.

27. Actinomycosis—C. A. Cary, D. V. M., B. S., Auburn, Ala. Leaders in discussion, Willis Jones, B. F. Rea.

28. Gonorrheal Metastasis—By W. L. Champion, M. D., Atlanta, Ga. Leaders in discussion, M. L. Boyd, J. W. Clark, F. M. Ridley, Jr.

29. The Importance of Investigating the Kidneys in Obscure Abdominal Pain—By Leon Watkins, M. D., Birmingham, Ala. Leaders in discussion, W. A. Selman, M. L. Boyd, Willis Jones.

30. Diseases of the Nasal Accessory Sinuses and Methods of Treatment—By Hugh M. Lokey, M. D., Atlanta, Ga. Leaders in discussion, W. G. Harrison, T. E. Mitchell, J. M. Baird.

31. Salpingitis—By T. Brannon Hubbard, M. D., Montgomery, Ala. Leaders in discussion, J. D. S. Davis, F. K. Boland.

32. Traumatic Septic Arthritis with Report of Cases—By H. S. Munroe, M. D., Columbus, Ga. Leaders in discussion, Michael Hoke, Hugh McCulloh.

33. Some Experiences of a Country Doctor—By C. S. Yarbrough, M. D., Beulah, Ala. Leaders in discussion, A. L. Harlan, T. H. Haralson.

34. A New Treatment for Burns—By Henry R. Slack, M. D., LaGrange, Ga. Leaders in discussion, J. S. Horsley, J. H. McDuffie.

Officers of the Chattahoochee Valley Medical and Surgical Association: President, Dr. O. V. Langley, Opelika, Ala.; Vice-President, Dr. H. Stokes Munroe, Columbus, Ga.; Secretary-Treasurer, Dr. W. J. Love, Opelika, Ala.

Board of Council: President, Dr. J. H. McDuffie, Columbus, Ga.; Dr. A. L. Harlan, Alexander City, Ala.; Dr. M. L. Boyd, Atlanta, Ga.; Dr. George H. Cooper, Opelika, Ala.; Dr. Hugh McCullough, West Point, Ga.

Committee on Entertainment: Chairman, Dr. I. W. Ballard, Opelika, Ala.; Dr. M. D. Thomas, Opelika, Ala.; Dr. G. H. Moore, Opelika, Ala.; Dr. Geo. H. Cooper, Opelika, Ala.

---

A strong solution of potassium permanganate painted over macerated, sodden skin surfaces, as between the toes or about the anus, and allowed to dry, gives much relief and often effects a cure. The application may be made daily or less frequently. Keep the painted surface dry with talcum powder and absorbent cotton.—American Journal of Surgery.

